# Exploring the Theoretical Foundation with Rupture and Delayed Rupture Experiments

**DOI:** 10.1021/acs.macromol.5c03203

**Published:** 2026-02-18

**Authors:** Asal Y Siavoshani, Ming-Chi Wang, Cheng Liang, Aanchal Jaisingh, Junpeng Wang, Chen Wang, Shi-Qing Wang

**Affiliations:** a School of Polymer Science and Polymer Engineering, 1076 University of Akron, Akron, Ohio 44325, United States; b Department of Materials Science and Engineering, 43334 University of Utah, Salt Lake City, Utah 84112, United States

## Abstract

We carry out uniaxial continuous and step stretching of various cross-linked polymer networks to demonstrate how characteristics of rupture during continuous stretching and delayed rupture after step stretching can be used to probe the structure of the emergent kinetic activation theory of bond dissociation (KATBD) for elastomeric failure. Based on delayed rupture experiments, we show that the network lifetime *t*_ntw_, taken as the incubation time *t*_del‑rupt_ for delayed rupture, depends on temperature in an Arrhenius like manner and is exponentially sensitive to the degree of network stretching (depicted by the step-stretch ratio λ_ss_). Rupture at λ_b_ during continuous stretching for a wide range of stretch rates takes place on time scales inversely proportional to the stretch rate. The elapsed time *t*_rupt_ at rupture is found to be comparable to *t*_del‑rupt_ at various values of λ_b_ = λ_ss_ in a wide range of temperature, affording the experimental basis for the premise of the KATBD. Having identified the hidden internal clock *t*_ntw_, continuous stretching tests at different temperatures are performed to show the existence of a new time temperature equivalence (TTE): fast stretching at higher temperatures is equivalent to slow stretching at lower temperatures: different pairs of rate and temperature can produce the rupture at the same tensile strength and strain.

## Graphical abstract

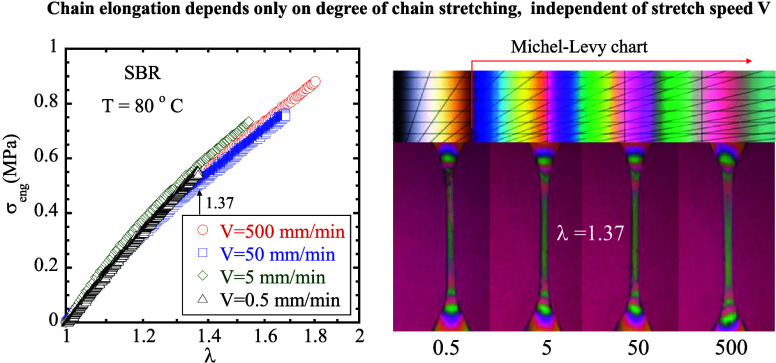

## 1 Introduction

Elastomeric rupture, resulting from continuous stretching of unnotched specimens until sudden macroscopic separation, has been treated in the literature as a fracture mechanical phenomenon by assuming the pre-existence of sizable flaws. Consequently, instead of performing an extensive illustration of rupture characteristics following the pioneering investigations of Smith and others,^1–3^ nearly all studies in the past^4–7^ have focused on fracture behavior of prenotched systems. Recent spatially temporally resolved polarized optical microscopic (*str-*POM) measurements of several cross-linked rubbery polymers showed^8,9^ that the tip birefringence upon visible crack propagation is comparable to that observed at rupture of unnotched polymers. Therefore, elastomers may be generally flaw insensitive, and rupture may be treated and understood outside the scope of fracture mechanics: The characterization of rupture in terms of tensile strength and rupture strain indeed involves no concepts such as energy release rate from fracture mechanics; on the other hand, both rupture and fracture (i.e., crack growth in a prenotched elastomer) should be controlled by bond dissociation. Thus, we can build our understanding of elastomeric fracture through a detailed study of elastomeric rupture on different time scales and at different temperatures.

Temperature controls the mechanical behavior of polymeric materials in several crucial ways. In polymer processing, there is the well-known time–temperature equivalence (TTE_ve_) associated with viscoelasticity,^10^ dictating the rheology of polymers in liquid (molten or rubbery) states.^11,12^ Here, Williams, Landel, and Ferry (WLF) found^10^ polymer viscosity and chain relaxation time τ_ve_ to explicitly vary with temperature. It has since been well-established that slower relaxation dynamics at lower temperatures is associated with higher viscosity. For seven decades,^13,14^ researchers advocated^15–20^ that the same viscoelastic processes dictated time and temperature dependencies of rupture and fracture in elastomers. This viewpoint has remained unchallenged until recently,^21^ even though the WLF shift did not produce^22^ any clear explanation for the observed temperature dependence of tensile strength at rupture and energy release rate for crack growth.

A different time–temperature equivalence (TTE_ntw_), hidden for seven decades, has recently been suggested^21^ to characterize elastomeric rupture and fracture, where the subscript “ntw” stands for the network lifetime *t*_ntw_ associated with bond dissociation in highly stretched elastomers. The lifetime *t*_ntw_ of the chain network is the hidden internal clock in the elastomers. A network, composed of permanent chemical cross-links and transient physical cross-links due to interchain uncrossability,^23^ can disintegrate when a percolating fraction of load bearing strands (LBS) undergoes chain scission. Determined by bond dissociation in backbones of LBS in high tension produced by stretching at (nominal) stretch ratio λ, *t*_ntw_ depends on temperature *T* in an Arrhenius manner, e.g., showing an exponential dependence on reciprocal temperature 1/*T*. Moreover, we expect *t*_ntw_(λ, *T*) to vary with the network structure that may be crudely characterized in terms of cross-link density.

Built on earlier treatments that regard overcoming solid strength as an activated process,^24,25^ the recent kinetic theory of bond dissociation (KATBD) for rupture^21,26^ asserts that rupture occurs because the elapsed time until rupture, given by

$$t_{\mathrm{rupt}}=\left(\lambda_{b}-1\right)/\mathrm{\lambda ̇}$$ 1

approaches *t*_ntw_, as illustrated in Figure 1. In other words, continuous stretching of an unnotched elastomer until rupture produces a measure of the network lifetime *t*_ntw_. Here, for simplicity, we express *t*_ntw_ in an Arrhenius-like form

$$t_{\mathrm{ntw}}(T,\lambda )=t_{\mathrm{ntw}0}(T)\exp[E_{\mathrm{ntw}}(\lambda )/RT]$$ 2

which also rapidly decreases with increasing λ, since the energy barrier *E*_ntw_ for network breakdown is a decreasing function of the level of network stretching. Equation 2 is clearly an oversimplification of reality. A more detailed description is currently unavailable.

**Figure 1:**
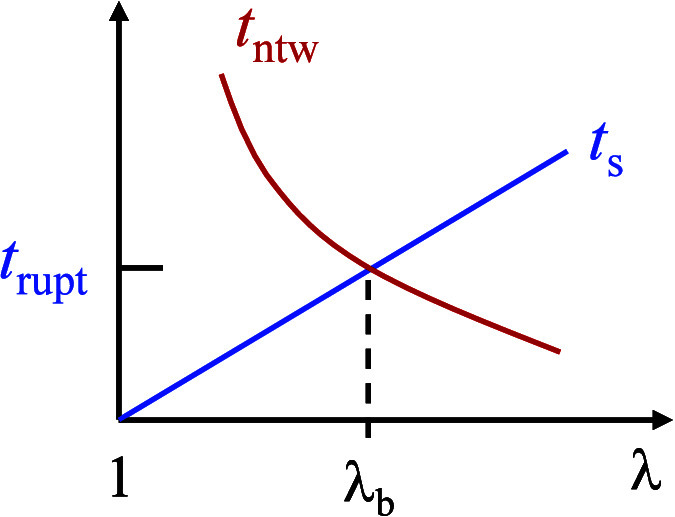
A schematic, illustrating a criterion for elastomeric rupture at stretch rate λ̇: The straight line represents the elapsed time of stretching, which increases with the stretch ratio λ. The curved line represents the network lifetime (*t*_ntw_), an intrinsic material property that decreases with the degree of network stretching. Rupture occurs at the intersection when the stretch time *t*_s_ increases to *t*_rupt_ of eq 1, i.e., increasing to the network’s lifetime (*t*_ntw_).

Delayed rupture has also been demonstrated to take place after an incubation time *t*_derupt_ following a step stretch. The pioneering study^3^ of Smith and Stedry revealed *t*_del‑rupt_(*T*, λ_ss_) to explicitly depend on temperature *T* and the degree of step stretching (ratio λ_ss_). This phenomenon has been treated in terms of the prevailing framework for polymer viscoelasticity.^14^ Our analysis and observation contradict this understanding. We suggest that *t*_del‑rupt_ reflects the network lifetime, i.e., *t*_del‑rupt_(*T*, λ_ss_) = *t*_ntw_(*T*, λ_ss_) of eq 2. It follows from the KATBD upon equating eq 1 with eq 2 that *t*_del‑rupt_(*T*, λ_ss_) = *t*_rupt_(*T*, λ_b_) at λ_b_ = λ_ss_.

One objective of the present study is to offer a fundamentally different interpretation of the nature of delayed rupture in contrast to the assertion^17^ that “the time consumed (for elastomers) in reaching the critical condition for bond decomposition” is far greater than the time required for bond decomposition, a conclusion^15,17^ that originated from Bueche and Halpin. To separate the various time scales, we carried out *in situ* birefringence measurements to contradict this flawed paradigm and suggest that delayed rupture in elastomers is not governed by chain dynamics but results from chain scission – an activated process^21^ through which network rupture takes time to materialize.

Moreover, in the elastic stretching limit,^23^ TTE_ntw_ governs rupture: Rupture will occur at the same value of λ_b_ at two different temperatures when two (non-Hencky) stretch rates λ̇_1_ and λ̇_2_ are chosen to be the reciprocal of the lifetime *t*_ntw_ at the two respective temperatures. According to eq 1 and *t*_rupt_ *= t*_ntw_, the condition is λ̇_1_ *t*_ntw_(λ_b_, *T*_1_) = λ̇_2_ *t*_ntw_(λ_b_, *T*_2_).

In this work, we perform continuous and step stretching of several elastomeric systems along with *in situ* birefringence to investigate rupture and delayed rupture at various temperatures, involving different stretch rates and different magnitudes of step stretching (characterized by λ_ss_). Quantitative characterizations allow us to show (a) how lifetime *t*_ntw_(λ, *T*) changes with the level of stretching (λ) and temperature (*T*) and (b) that faster stretching at higher temperatures is equivalent to slower stretching at lower temperatures, i.e., there exists TTE_ntw_, involving *t*_ntw_ as the pertinent time scale. In conclusion, (1) delayed rupture may be understood in terms of chain scission instead of polymer viscoelasticity, (2) network lifetime varies with temperature in a manner distinctly different from the WLF temperature dependence, and (3) TTE_ntw_ emerges to show that faster stretching to produce rupture at higher temperatures is equivalent to slower stretching at a lower temperature.

## 2 Experimental Section

### 2.1 Sample Preparation

The materials used in this study were a commercial acrylate-based elastomer, VHB 4910 (3 M product), two cross-linked styrene butadiene rubber (SBR0.1 phr and SBR0.03 phr), a cross-linked poly­(methyl acrylate) (×PMA), and a polyamide elastomer.

The SBR elastomers (SBR0.03 phr and SBR0.1 phr) were prepared by peroxide-initiated cross-linking. A masterbatch was created by dissolving 100 g of SBR and 0.03 and 0.1 g of dicumyl peroxide (DCP) cross-linker for SBR0.03 phr and for SBR0.1 phr, respectively, into 400 mL of an appropriate solvent (e.g., toluene). The mixture was stirred for 72 h to ensure homogeneity. Following mixing, the solvent was removed by drying the solution in a vacuum oven at 70 °C for 24 h. The resulting rubber compound was then cured into sheets using a hot press at 150 C for 20 min. Finally, tensile test specimens were cut from the cured sheets using a homemade dogbone-shaped die cutter with a gauge section measuring 9 mm in length, 1.56 mm in width, and an average final thickness of 1.3 mm. The dogbone-shaped specimens were placed between the clamps at a distance of *L*_0_ = 17 mm (cf. inset of Figure 4a).

For the synthesis of the cross-linked poly­(methyl acrylate) (PMAx) elastomer, a precursor solution was first prepared. Methyl acrylate (110 mmol, 100 equiv) was combined with an equal volume of chloroform. Butanediol diacrylate (1.1 mmol, 1 equiv) as a cross-linker and Irgacure 819 (0.11 mmol, 0.1 equiv) as a photoinitiator were then dissolved in the mixture. The solution was deoxygenated via a 20 min nitrogen purge. Under a nitrogen atmosphere, the solution was transferred to a glass-silicone-glass mold (120 × 120 × 1.4 mm). Curing was achieved by irradiating the sample with 365 nm UV light for 1 h. The resulting film was removed and purified by immersion in toluene for 24 h, during which the solvent was refreshed three times to remove the sol fraction. Finally, the film was deswollen in methanol, air-dried for 1 h, and dried under high vacuum at 50 °C for 24 h. From these finished sheets, tensile test specimens were die cut into dogbone geometry. The specimens have a gauge section measuring 9.53 mm in length and 3.18 mm in width and an average final thickness of 1.3 mm. The dogbone shaped specimens were placed between the clamps at a distance of *L*_0_ = 27 mm (cf. inset of Figure 4b). VHB was also cut into such a dogbone shape (cf. inset of Figure 4c).

For the synthesis of the polyamide elastomer, the diallyl *m*-phthalamide monomer was synthesized as previously reported.^27^ To prepare one dog-bone specimen for the fracture analysis, 0.49 g of TEMPIC (0.9 mmol) and 0.34 g of EBMP (1.4 mmol) were predissolved in a scintillation vial using the speed-mixer for 1 min at 3500 rpm. To this dithiol and trithiol mixture, the synthesized diallyl *m*-phthalamide monomer (2.7 mmol) and 15 mg of TPO-L were added. This was followed by gentle heating to melt and mix the contents of the reaction mixture. The resin was then poured into the preheated silicon mold at 70 °C and subjected to UV-curing using a dogbone shaped mold with a gauge section measuring 9.53 mm in length and 3.18 mm in width, with an average final thickness of 1.3 mm with the clamping distance of 27 mm at 405 nm at 70 °C for 3 min.

### 2.2 Methods

Mechanical testing of the VHB, SBR, and ×PMA samples was conducted using an Instron 5969 universal testing machine equipped with a temperature controlled environmental chamber. Samples were mounted by using two clamps to perform continuous and stepwise tensile stretching. Some continuous and delayed rupture tests were video-recorded deformation was recorded using a 4K camera (Mokose) fitted with a 5–100 mm zoom lens (Arducam).

Continuous uniaxial tensile tests were performed on the SBR samples (with gauge length *L*_0_ = 17 mm) to investigate the temperature and rate effects. The experiments were conducted at four different temperatures: room temperature (RT, ca. 25 °C), 50 °C, 70 °C, and 80 °C. At each temperature, a range of crosshead speeds were selected to achieve specific nominal strain rates (*V*/*L*_0_), allowing for the comparison with delayed rupture on different time scales. The specific stretch rate, *V*/*L*_0_, investigated at each temperature were as follows: RT and 80 °C: ca. 0.0005, 0.005, 0.05, and 0.5 s^–1^ (corresponding to *V* = 0.5 to 500 mm/min), 50 °C: ca. 0.0005, 0.005, 0.1, and 0.5 s^–1^, and 70 °C: 0.001, 0.014, and 0.5 s^–1^. This experimental protocol was designed to identify the stretch rate required to achieve a targeted range of times for rupture at each temperature, enabling a systematic demonstration of the new time–temperature equivalence. The results are presented in Figure 8a,b.

## 3 Results and Discussion

### 3.1 *In Situ* Birefringence to Elucidate the Nature of Rupture and Delayed Rupture

We first carried out *in situ* birefringence measurements to show that SBR0.1 phr exhibits the same degree of average chain elongation independent of the stretch rate used to perform continuous stretching. Figure 2a shows the stress response to be independent of the stretch rate. At the same stretch ratio, the birefringence is also the same, as shown in Movies 1 and 2 and in Supporting Information (SI). Figure 2b contains four identically looking birefringence images at the same strain of λ – 1 = 0.37. Such results remove Bueche’s confusion related to his creep tests^15^ that “the strength of a rubber should be much greater under a fast than a slow test since in the former case the network chains are not able to elongate very far during the period of the test”: In displacement-controlled stretching, chain elongation is affinity-like so that birefringence resulting from the chain orientation is the same, independent of stretching time (stretch rate) required to produce the same nominal strain.

**Figure 2:**
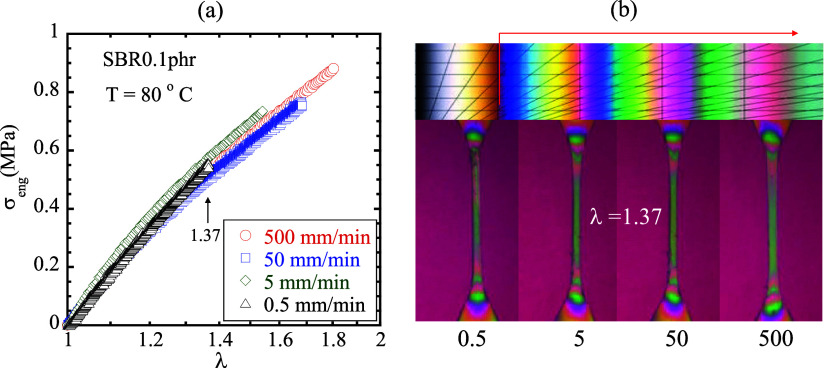
(a) Engineering stress versus stretch ratio λ for SBR0.1 phr with *L*_0_ = 17 mm at 80 °C, stretched with crosshead speeds *V* = 0.5, 5, 50, and 500 mm/min. (b) Birefringence images at λ = 1.37 from all four continuous stretching tests, where 1.37 is λ_b_ for *V* = 0.5 mm/mm, as indicated by the last triangle in (a). According to the Michel-Levy chart, specimens reached the fourth green at λ = 1.37 in the presence of a retardance plate that produces the first red as background.

Motivated by KATBD, we then perform continuous stretching at *V* = 5 mm/min and observe rupture at λ_b_ = 1.53. Subsequently, we carry out a step stretch to λ_ss_ = 1.5 ∼ λ_b_ using a high rate, e.g., *V* = 200 mm/min. Both continuous and step stretching are carried out along with *in situ* birefringence observations in the form of Movies 3 and 4. Figure 3a shows the stress vs strain curves of these two experiments, confirming the preceding conclusion that stress and chain elongation (shown in Figure 3b) are both independent of stretch rate. Replotting Figure 3a, we show in Figure 3c that elapsed time *t*_rupt_, given by eq 1, at rupture in continuous stretching is of the same magnitude as the incubation time *t*_del‑rupt_ for delayed rupture. Since *t*_del‑rupt_ increases rapidly with decreasing λ_ss_, we can readily separate the polymer relaxation time τ from the incubation time. When *t*_del‑rupt_ ≫ τ, delayed rupture cannot be explained using the previous paradigm^14^ of polymer viscoelasticity-energy dissipation; during the incubation period, there is no ongoing stretching and there is little change in the birefringence. The concealed internal clock that dictates when delayed rupture occurs is the network lifetime *t*_ntw_.

**Figure 3:**
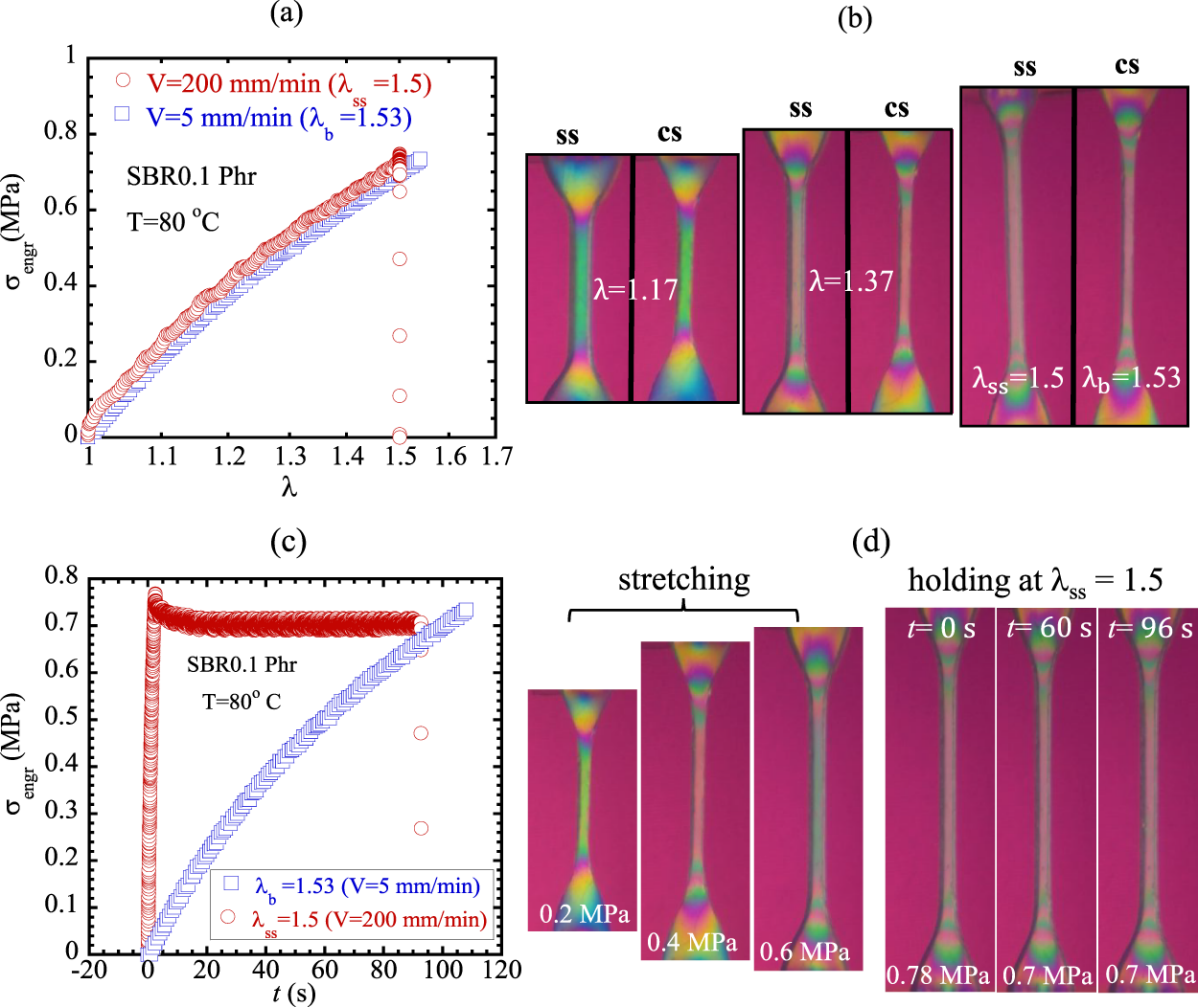
(a) Uniaxial stretching of SBR0.1 phr (*L*_0_ = 17 mm) at 80 °C under respective continuous (with *V* = 5 mm/min in circles until rupture at λ_b_ = 1.53) and step (with *V* = 200 mm/min to hold at λ_ss_ = 1.5 in squares) stretching conditions. (b) Corresponding birefringence images comparing chain elongation at common stretch ratios of λ = 1.17, 1.37, and ca. 1.5. (c) Replot of (a) in the time domain. (d) Birefringence images at various stages of step stretch to λ_ss_ = 1.5 as well as the last three nearly identical images during the stress relaxation.

Since SBR0.1 phr is an elastomer, the level of chain elongation remains essentially unchanged after the step stretch, as confirmed by *in situ* birefringence observations in Figure 3d. This result contradicts the assertion of Smith and Stedry^3^ that originated from the claim of Bueche^15^ and Halpin^17^ that elastomers take most of their time to creep to critical chain elongations for bond decomposition and the time required for decomposition is insignificant by comparison. It also contradicts any explanation based on energy dissipation or polymer viscoelasticity^14^ since there is no ongoing stretching to produce any viscous flow – remarkably, Kinloch^14^ did not cite this seminal work of Smith and Stedry^3^ in his book. Thus, for the first time, we assert that delayed rupture occurs not because step stretch produces chain elongation that could grow after step stretch but because bond dissociation as activated events takes time to produce a path of chain scission in the network, leading to rupture across the width of a macroscopic specimen. Specifically, we interpret the incubation time *t*_del‑rupt_ for delayed rupture as the network’s lifetime *t*_ntw_(λ_ss_, *T*). In support of the KATBD, *t*_del‑rupt_ is on the order of *t*_rupt_ so that we may draw the conclusion that *t*_del‑rupt_ (λ_ss_,*T*) = *t*_ntw_ ∼ *t*_rupt_(λ_b_,*T*), as shown in Figure 1.

The behavior of SBR0.1 phr shown in Figure 3c,d appears to be universal. In the limit of elastic stretching until rupture and delayed rupture, other elastomers also show such behavior in Figure 4a–c, based on SBR0.03 phr at 25 °C, ×PMA at 40 °C, and VHB at 90 °C. We will summarize the comparison of time scales involved in rupture and delayed rupture in Section 3.4.

**Figure 4:**
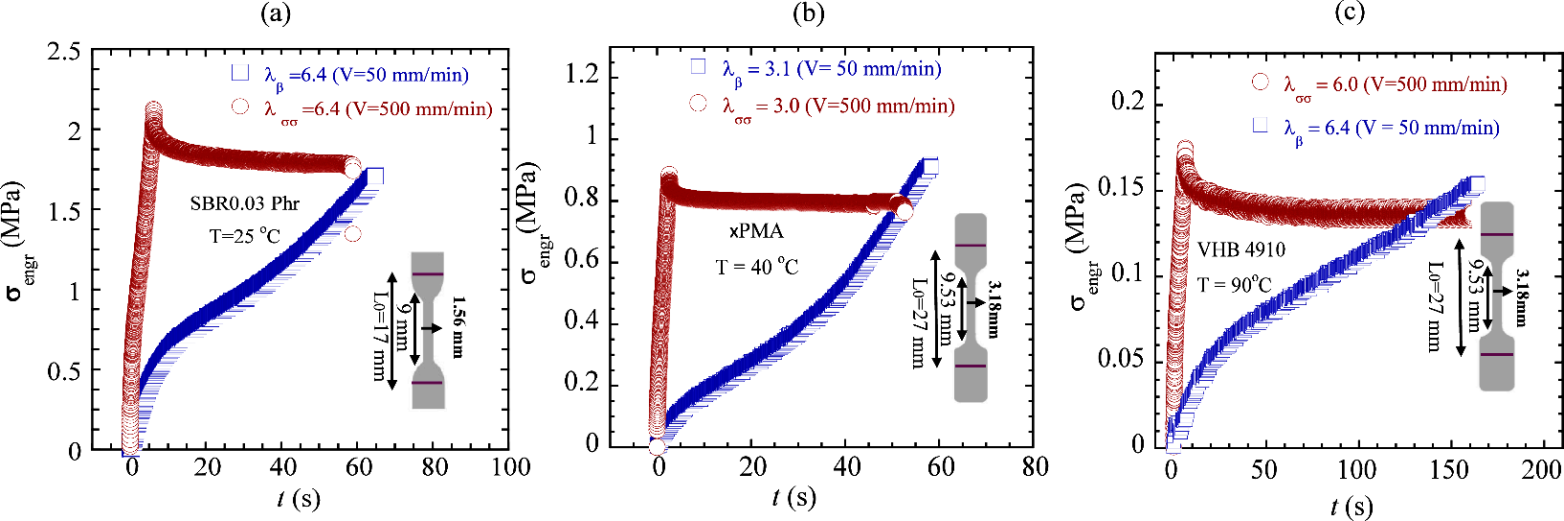
Engineering stress vs time under respective continuous and step stretching for (a) SBR0.03 phr (*L*_0_ = 17 mm) at room temperature, (b) ×PMA (*L*_0_ = 27 mm) at 40 °C, and (c) VHB (*L*_0_ = 27 mm) at 90 °C, where λ_b_ characterizes the rupture strain and λ_ss_ designates the magnitude of the step stretch.

### 3.2 Pertinent Time Scale Revealed by Delayed Rupture: Temperature Dependence

Delayed rupture after step stretching explicitly reveals the hidden internal clock in elastomers. By carrying out step stretching at different temperatures, we measure how this new time scale depends on temperature. Specifically, the incubation time taken for a step-stretched specimen to undergo sudden delayed rupture, *t*_del‑rupt_, reflects the network’s lifetime *t*_ntw_, as given by eq 2. Figure 5a shows that *t*_del‑rupt_ is approximately Arrhenius, i.e., exponentially varying with 1/*T*. More importantly, the inset shows that this temperature dependence systematically deviates from the WLF dependence (solid line). The deviation can be either positive or negative, with *t*_del‑rupt_ showing either a stronger or weaker temperature dependence than that of the WLF shift factor *a*_T_. For example, unlike VHB, vulcanized SBR exhibits positive deviation, as shown in Figure 5b, consistent with the first study^3^ of Smith and Stedry whose Figure 8 also showed^26^ a stronger temperature dependence than that prescribed by the WLF factor *a*_T_. Since *t*_del‑rupt_ reflects the elastomer’s lifetime *t*_ntw_ of eq 2, the deviation of *t*_del‑rupt_ from the WLF temperature dependence strongly suggests that the internal clock governing elastomeric failure is not polymer relaxation time.

**Figure 5:**
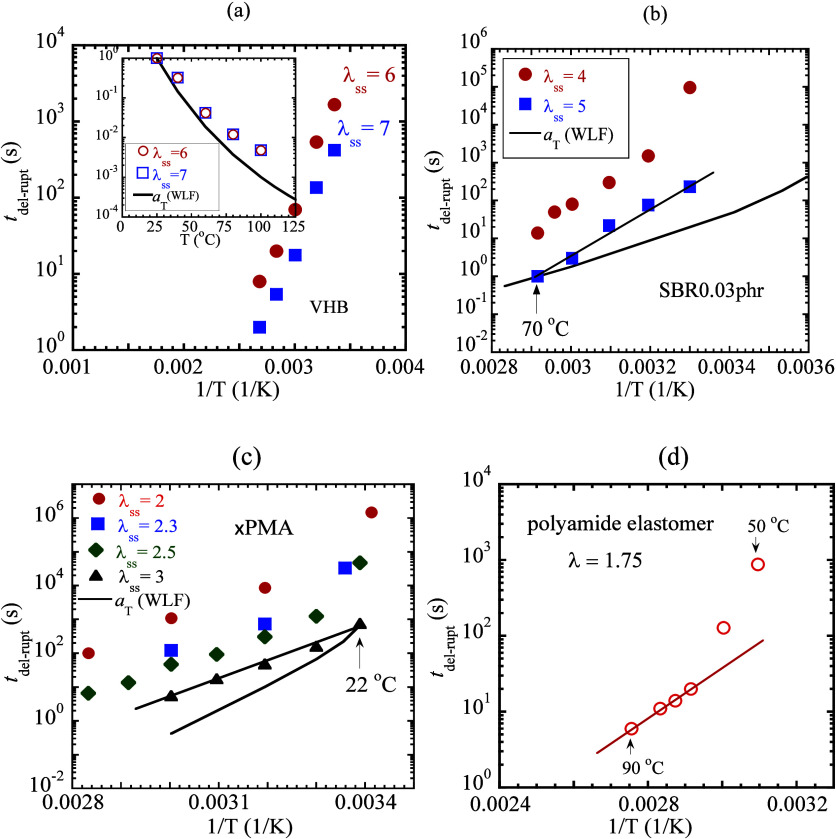
Dependence of network lifetime *t*_ntw_ on temperature, taken as the incubation time *t*_del‑rupt_ for delayed rupture of four different elastomers after step stretching using a crosshead speed of 500 mm/min: (a) VHB (*L*_0_ = 27 mm) held at λ_ss_ = 6 and 7, where the inset figure directly compares the temperature dependence of WLF shift factor *a*_T_ with that of *t*_del‑rupt_, (b) SBR0.03 phr (*L*_0_ = 17 mm) held at λ_ss_ = 4 and 5, where the smooth curve is the WLF shift factor, (c) ×PMA (*L*_0_ = 27 mm) held at λ_ss_ = 2, 2.3, 2.5, and 3, where the smooth curve is the WLF shift factor, and (d) polyamide elastomer (*L*_0_ = 27 mm) held at λ_ss_ = 1.75.

Figure 5b also reveals an interesting feature for λ_ss_ = 4. The last data point at room temperature appears to show an upward deviation from other data points that form a straight line (Arrhenius dependence). The off-Arrhenius behavior is not unique to SBR. For verification, ×PMA is step-stretched at different values of λ_ss_. In addition to the negative departure from the WLF temperature dependence, we observe *t*_del‑rupt_ to show a notable deviation from Arrhenius behavior at the lowest test temperatures, as shown in Figure 5c. To confirm this trend, a fourth polyamide based elastomer^27^ (a thermoset at elevated temperatures) is studied in step-stretch tests and shown in Figure 5d to reveal a similar upward deviation. This deviation only occurs at lower temperatures where reduced chain mobility may start to play a non-negligible role to slow the formation of the physical path, along which delayed rupture takes place. Further investigation in this direction is beyond the scope of the current study.

Having conducted continuous stretching tests,^1^ Smith and Stedry anticipated^3^ delayed rupture perhaps because they thought that their SBR just needed more time after a fast step stretching for chains to achieve critical molecular strain for chain decomposition. For example, in Figure 3a, it took ca. 100 s for rupture (squares) to occur, and the step stretch only took 2.55 s. Therefore, according to Smith and Stedry,^3^ we should wait for about 97 s for delayed rupture to take place. This 97 s was understood by Bueche,^15^ Halpin,^17^ Smith,^3^ and most others to be required for molecular strain to be established for chain decomposition. However, our birefringence measurements in Figure 3b indicate that the molecular strain is already achieved in 2.55 s. According to our KATBD, the stretched network spends these 100 s at this stretch ratio of 1.5, waiting for bond dissociation to destroy the network.

### 3.3 Solvent Effect on the Network Lifetime

Solvent effects have been suggested to minimize polymer viscoelasticity and reduce energy dissipation.^20,28^ Rubber swelling by solvent was used to access the fatigue threshold.^29,30^ In the KATBD description of the structural origin of elastomeric failure,^21^ we suggest that solvent may accelerate the network lifetime. In other words, the increased chain mobility might have shortened the time for the network to “search” for the failure path. To demonstrate the solvent effect, we incorporate less than 10% of dibutyl adipate into VHB and 10% of dimethyl sulfoxide into ×PMA so that their elastic responses and stress relaxation behavior are little affected, as shown in SI.1a-b in the Supporting Information (SI). Figure 6a,b indicates that the incubation time *t*_del‑rupt_ is shortened by a decade in the presence of solvent for both VHB and ×PMA, where circles represent the *t*_del‑rupt_ of solvent-free elastomers.

**Figure 6:**
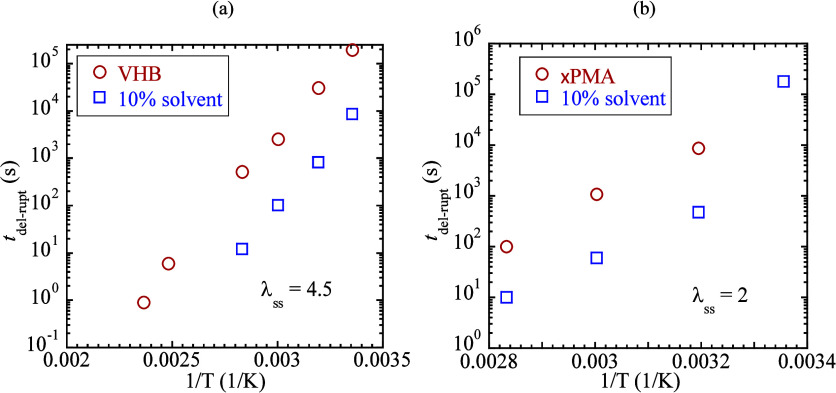
Solvent effect on delayed rupture: (a) incubation time *t*_del‑rupt_ for delayed rupture of (a) of VHB (*L*_0_ = 27 mm) at various temperatures and (b) PMA­(*L*_0_ = 27 mm), both shorter by a decade by the solvents (squares).

### 3.4 Comparison of Two Time Scales

Stretching with rate λ̇ produces rupture on atime scale (*t*_rupt_) approximately inversely proportional to λ̇ per eq 1. In other words, given the explanation of Figure 1, characteristics of rupture such as *t*_rupt_ and λ_b_ can be used to reveal the network lifetime over a range as wide as that of the applied rate. Figure 2a, along with Figure SI.2a–c (VHB, SBR, PMA), shows that with increasing stretch rate, rupture occurs at measurably higher λ_b_. Since strain (λ_b_ – 1) at rupture only logarithmically increases with λ̇, applied rate λ̇ approximately discloses the network lifetime per eq 1.

To measure network lifetime *t*_ntw_, we consider step-stretching to different magnitudes, characterized by stretch ratio λ_ss_ by using the highest available stretch speed. For a range of λ_ss_, we measure *t*_del‑rupt_(λ_ss_) as shown in circles in Figure 7a,c,d, based on three different elastomers. The corresponding raw data are given as Figures SI3a-c in Supporting Information. Figure 7b indicates the relationship between the tensile strength and rate. In squares, Figure 7a,c,d shows *t*_rupt_ as a function of λ_b_ from corresponding continuous stretching tests with stretch rates λ̇ = *V*/*L*_0_ = 0.0005, 0.005, 0.05, and 0.5 s^–1^ for SBR0.1 phr, λ̇ = 0.0031, 0.031, and 0.31 s^–1^ for VHB, and λ̇ = *V*/*L*_0_ and λ̇ = *V*/*L*_0_ = 0.00031, 0.0031, 0.031, and 0.31 s^–1^ for ×PMA, where the ellipses mark the three separate cases where the raw data have been presented in Figures 2c and 4b,c, respectively.

**Figure 7:**
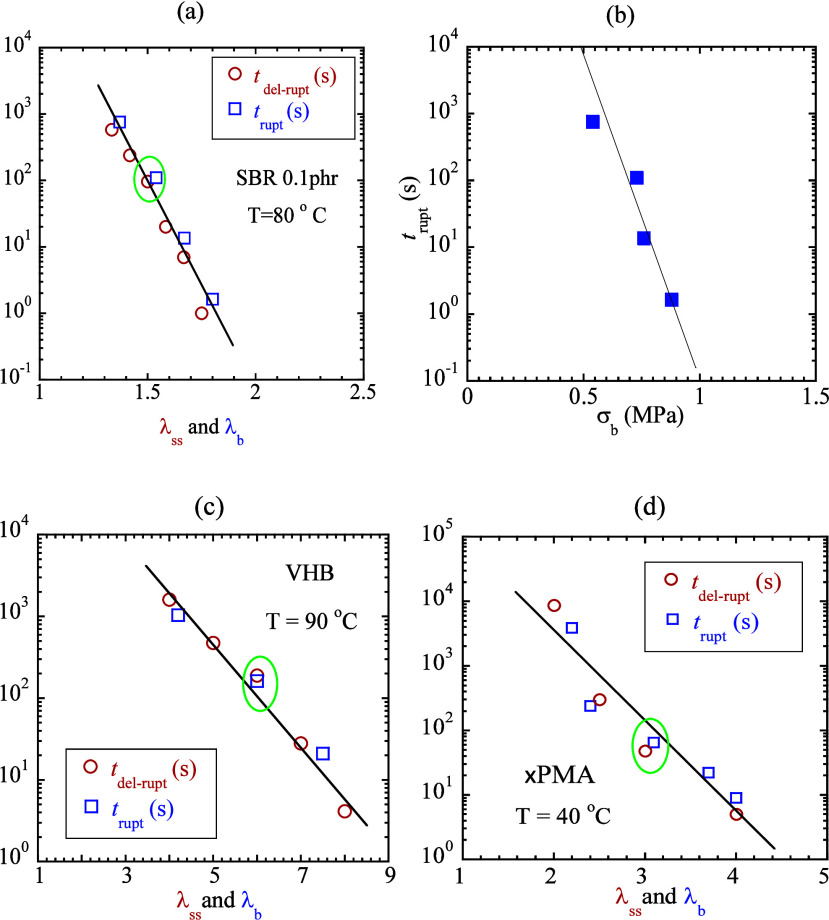
Two time scales, *t*_del‑rupt_ and *t*_rupt_, obtained from step and continuous stretching involving various step-stretch ratios λ_ss_ and rupture stretch ratios λ_b_, based on (a) SBR0.1 phr (*L*_0_ = 17 mm) at 80 °C, (b) *t*_rupt_ as a function of tensile strength σ_b_, (c) VHB (*L*_0_ = 27 mm) at 90 °C, and (d) ×PMA (*L*_0_ = 27 mm) at 40 °C.

The overlap of circles (*t*_del‑rupt_) and squares (*t*_rupt_) over several decades reveals that *t*_del‑rupt_(*T*, λ_ss_) = *t*_rupt_(*T*, λ_b_) at λ_ss_ = λ_b_. Since *t*_del‑rupt_ may be taken as *t*_ntw_, the overlapping in Figure 7a,c,d confirms the underlying physics illustrated in Figure 1: *t*_rupt_ = *t*_ntw_, i.e., rupture occurs when elastomers’ lifetime becomes comparable to the stretch time. In other words, in the elastic stretching limit, rupture tests also reveal the lifetime. We conclude that the network lifetime *t*_ntw_ is dictated by the degree of stretching, i.e., λ_ss_ for delayed rupture in the step stretch and by λ_b_ for rupture in the continuous stretch.

### 3.5 Time–Temperature Equivalence in Rupture

Like the polymer relaxation time, the network lifetime is also temperature dependent (cf., eq 2), as shown in Figure 5a–d. In the rheology of un-cross-linked melts,^12^ faster deformation at higher temperatures is equivalent to slower deformation at lower temperatures. Analogously, since changing temperature changes the network lifetime, which dictates when rupture occurs, there is new time–temperature equivalence. Faster stretching at a higher temperature is equivalent to slower stretching at a lower temperature. This new TTE_ntw_ is based on the temperature dependence of lifetime *t*_ntw_(*T*) and has nothing to do with the familiar TTE_ve_ in polymer rheology that involves the chain relaxation time.

Because of TTE_ntw_, we can identify a pair of values for stretch rate λ̇ and temperature *T* that produces the same rupture strain, i.e., the same λ_b_. For this purpose, we carry out both continuous and step stretching of SBR0.03 phr at four temperatures to show four sets of data in Figure 8a, including the pair at 25 °C in the ellipse whose raw data have been already presented in Figure 4a. Raw data at other temperatures can be found in SI.4a-c. At 80 °C, λ_ss_ and λ_b_ falls in the range from 3 to 4.5, whereas the range is from 4 to 7.7 at 25 °C. The change in λ_ss_ and λ_b_ at different temperatures reflects the temperature dependence of *t*_ntw_: at lower temperatures, more stretching is required to produce the same lifetime.

**Figure 8:**
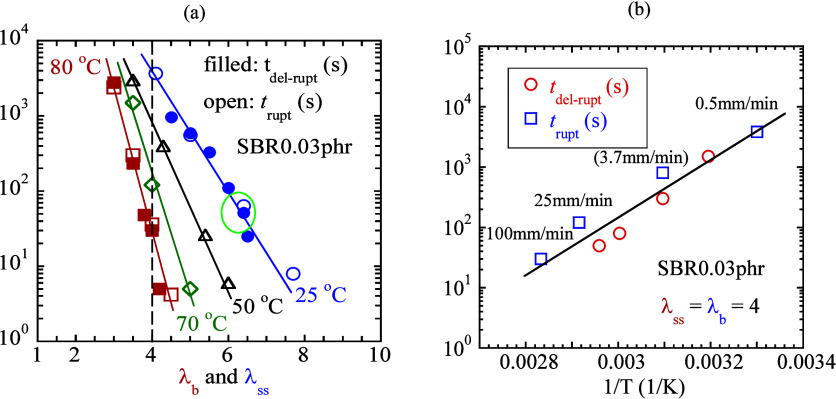
(a) Two time scales, *t*_del‑rupt_ and *t*_rupt_, obtained from step and continuous stretching involving various step-stretch ratios λ_ss_ and rupture stretch ratios λ_b_, based on SBR0.03 phr (*L*_0_ = 17 mm) at four temperatures of 25, 50, 70, and 80 °C. (b) New time–temperature equivalence (squares), confirmed by the temperature dependence of the network lifetime (circles). The same rupture at λ_b_ = 4 is produced at four different temperatures using four different stretch rates, where the value of 3.7 mm/min at 50 °C is read from the extrapolating straight line in (a).

Reading Figure 8a along the vertical dashed line, for example, at λ_b_ = 4, we show in Figure 8b that these rates produce rupture at different times given by *t*_rupt_ in squares as a function of temperature. The data of *t*_del‑rupt_ from delayed rupture in circles are from Figure 5b. The overlap confirms the implication as that in Figure 7a,c,d did: the *t*_rupt_ of eq 1 reflects the network lifetime *t*_ntw_. Figure 8a shows how the two time scales change with the degree of network stretching, whereas Figure 8b shows how they change with temperature.

In the temperature range from 25 to 80 °C, varying stretch rates from 5 × 10^–4^ to 0.5 s^–1^ can equivalently produce the same rupture, i.e., rupture at the same σ_b_ and λ_b_. In other words, Figure 8b reveals that rupture at λ_b_ = 4 in the range of temperatures from 25 to 80 °C involves a change in *t*_ntw_ = *t*_rupt_ (squares) of ca. two decades, produced by different stretch rates from 0.1 (80 °C) to 5 × 10^–4^ (25 °C) s^–1^. Such a demonstration of TTE_ntw_ has an added benefit: it automatically reveals the temperature dependence of *t*_ntw_ = *t*_rupt_, where the equality follows from the approximate collapses of squares and circles.

Finally, we indicate that the tensile strength systematically increases with applied rate, as shown in Figure 9. Here, since rupture occurs on shorter time scales at higher rates, it necessarily involves higher tensile strength – only higher strain can shorten the elastomer’s lifetime. At higher temperatures, smaller changes in chain stretching, reflected in tensile strength σ_b_, are involved to result in the same magnitude of change in the network lifetime. Consequently, we see the trend that the change of rate with tensile strength is sharper at higher temperatures. Figure 9 is in complete agreement with results^1,2^ in the literature. However, our interpretation of Figure 9 is fundamentally new.

**Figure 9:**
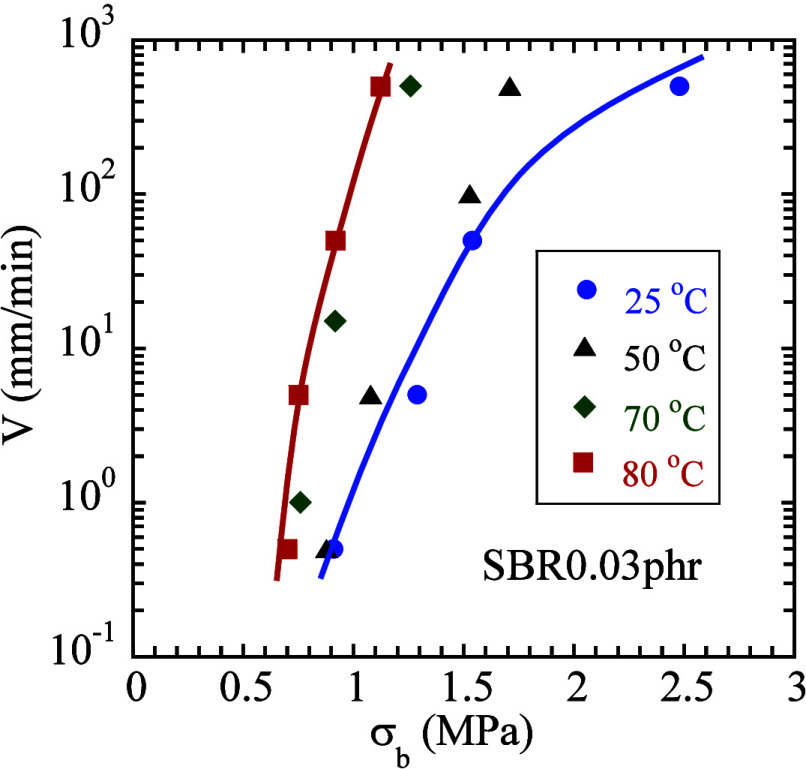
Tensile strength as a function of crosshead speed *V* of SBR0.03 phr (*L*_0_= 17) at four temperatures.

## 4 Conclusions

Guided by the central idea that bond dissociation in backbones of most stretched network strands controls characteristics of elastomeric rupture, we carried out continuous and step stretching under a wide range of stretch rates, aiming to verify the foundation of the KATBD. Two key ingredients or pillars of the KATBD are (a) network lifetime *t*_ntw_ exponentially decreasing with network stretching and (b) rupture taking place when *t*_ntw_ decreases during continuous stretching until *t*_rupt_. As described in the last paragraph of Section 3.2, the rheological and rheo-optical observations show that elastomers, extended in displacement-controlled stretching, consume little time to reach the critical condition of chain stretching for bond decomposition and spend most of the time waiting for bond dissociation to result in rupture.

The present study demonstrates the following key results in the limit of elastic stretching where stress response is rate independent (cf. Figure 2a). First, step stretch tests at various temperatures show how the incubation time for delayed rupture, *t*_del‑rupt_, depends (1) on temperature (cf. Figure 5a–d) and (2) on imposed strain in an exponential manner (cf. Figure 7a,c,d). Second, continuous stretch tests with various stretch rates at different temperatures disclose how stretch rates define the time window, through which elastomers’ lifetime *t*_ntw_ matches the elapsed time *t*_rupt_ at rupture. Third, comparison between *t*_del‑rupt_ and *t*_rupt_ as a function of imposed strain λ_ss_ or λ_b_ (cf. Figure 7a,c,d) shows that rupture is indeed a moment when elastomeric lifetime *t*_nwt_, which is measured by *t*_del‑rupt_, equals the elapsed time *t*_rupt_: Rupture occurs when the elastomer reached its lifetime. Fourth, there is new time–temperature equivalence (TTE_ntw_) based on the lifetime *t*_ntw_. Under elastic stretching conditions, where this new TTE_ntw_ holds, fast stretching at high temperatures is equivalent to slow stretching at low temperatures. Similarly, at a given temperature, faster stretching produces rupture on a shorter time scale at larger strain because only higher strain makes the elastomer’s lifetime shorter. These conclusions fundamentally deviate from the foundation of the existing paradigm^13,14^ regarding elastomeric failure. For example, it was stated by Smith^18^ that “the strength and extensibility of an elastomer depend on its overall viscoelastic properties, as reflected in the time (rate) and temperature dependence of stress-strain curves”.

When stress responses are rate dependent,^23,31^ due to transient cross-linking effects such as entanglement, elastomers become less stretchable at higher rates. However, it remains true that shorter network lifetimes at high rates require higher stresses. There are also cases where stretchability hardly changes with rate. For example, some hydrogels show little rate effect on stretchability and only a weak increase of its tensile strength with applied stretch rate.^32^ Here, the lack of the rate independence of stretchability is still accompanied by a sizable increase of tensile strength with rate – as expected, consistent with the basic notion of KATBD: The shorter lifetime encountered at higher rate requires a higher tensile strength, as shown in Figure 7b. Figure 9 contains the same message: a small variation in nominal stress corresponds to a decade of change in the stretch rate. Similar information at the chain level has emerged in the literature,^33^ supporting our claim concerning the molecular origin of rupture and delayed rupture in elastomers.^34^

## Supplementary Material











## Notes

Figure SI.1a,b detailing elastic responses and stress relaxation behavior of VHB with dibutyl adipate and ×PMA with dimethyl sulfoxide. Figure SI.2a–c illustrating the increase in rupture stretch ratio (λ_b_) with increasing stretch rate for VHB, SBR, and PMA. Figure SI.4a–c presenting raw data for SBR0.03 phr at various temperatures (MP4)
Movie 1 showing birefringence images at the same stretch ratio for SBR0.1 phr (MP4)
Movie 2 showing birefringence images at the same stretch ratio for SBR0.1 phr (MP4)
Continuous stretching is carried out along with in situ birefringence observations (MP4)
Step stretching is carried out along with in situ birefringence observations (PDF)
