# Supplementary material for: Exploring the Theoretical Foundation with Rupture and Delayed Rupture Experiments
Source: Macromolecules. 2026 Feb 18;59(5):2885–93. doi: 10.1021/acs.macromol.5c03203 (PMC12981016; doi:10.1021/acs.macromol.5c03203)
Supplement: Supplementary file 5 [file ma5c03203_si_005.pdf]

Exploring the theoretical foundation with rupture and delayed rupture experiments

Asal Siavoshani, Ming-Chi Wang, Cheng Liang, Aanchal Jaisingh, Junpeng Wang,

Chen Wang, Shi-Qing Wang

This supporting information (SI) provides a detailed account of additional experimental information in the form of figures, including data from step strain relaxation tests on both pristine and solvent modified elastomer samples (VHB and xPMA) at various temperatures. Furthermore, it presents continuous uniaxial stress-strain curves for VHB, xPMA, and SBR0.03 Phr elastomers, demonstrating the influence of crosshead speed on their rupture behavior. This SI also details the stress relaxation behavior of these three elastomers under different step-strain conditions. Finally, it includes stress-strain curves for SBR at various crosshead speeds and elevated temperatures, from which rupture times are evaluated as a function of temperature and stretch rate. Birefringence images in Figures 2b, 3b and 3d are extraced from Movies 1 to 4 listed below.

1.Movie SBR0.5: : Continous stretching of SBR0.1phr sample at 80 º C with crosshead speed of 0.5 mm/min speeded up by 10x.

2.Movie SBR500: Continous stretching of SBR0.1phr sample at 80 º C with crosshead speed of 500 mm/min speeded down by 10x.

3.Movie SBR5: Continous stretching of SBR0.1phr sample at 80 º C with crosshead speed of 5 mm/min breaking at λ=1.53 .

4.Movie SBR-SS-1.5: step strain test for SBR0.1phr using crosshead speed of 200 mm/min to reach λ=1.5 and holding till break.

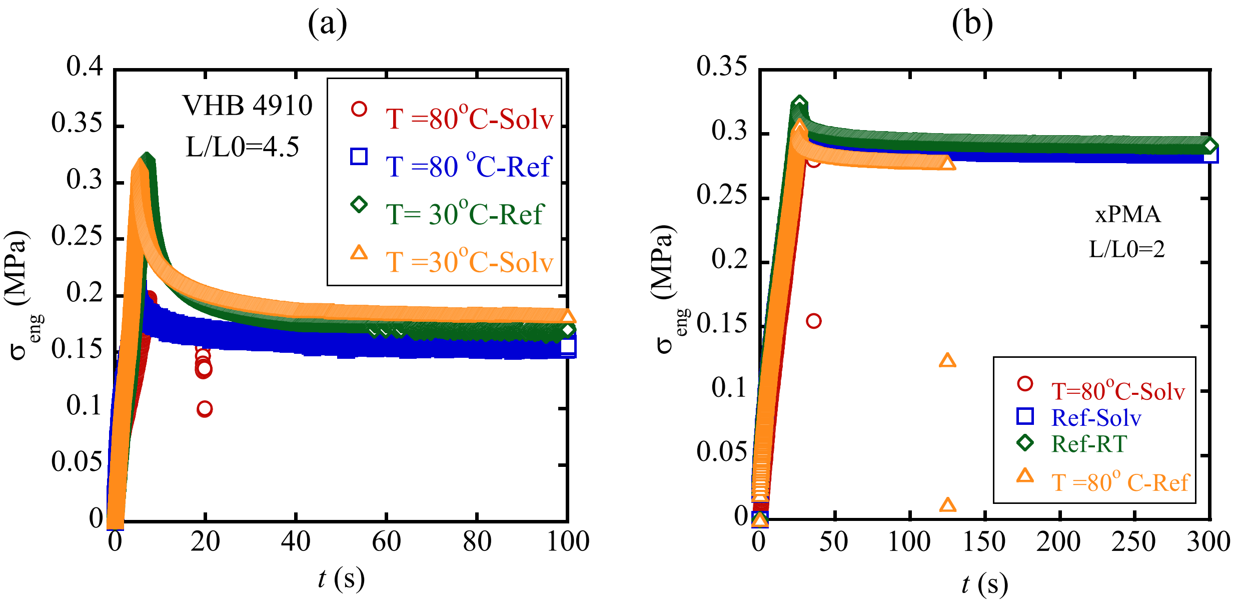

Fig. SI.1 Stress time curves from step strain relaxation tests on pristine and solvent modified elastomer samples. All dogbone specimens had nominal dimensions of 40 mm × 3.13 mm × 1 mm. (a) VHB samples were drawn to a strain of 4.5 at a crosshead speed of 500 mm min^{-1} and held at 30 °C and 80 °C. Both the pristine and solvent modified (10 wt% dibutyl adipate) samples display comparable stress levels during the isometric hold period. (b) Stress time behavior of xPMA samples, both pristine and containing 10 wt% dimethyl sulfoxide, tested at room temperature and 80 °C were drawn to a strain of 2 at a crosshead speed of 100 mm min^{-1}.

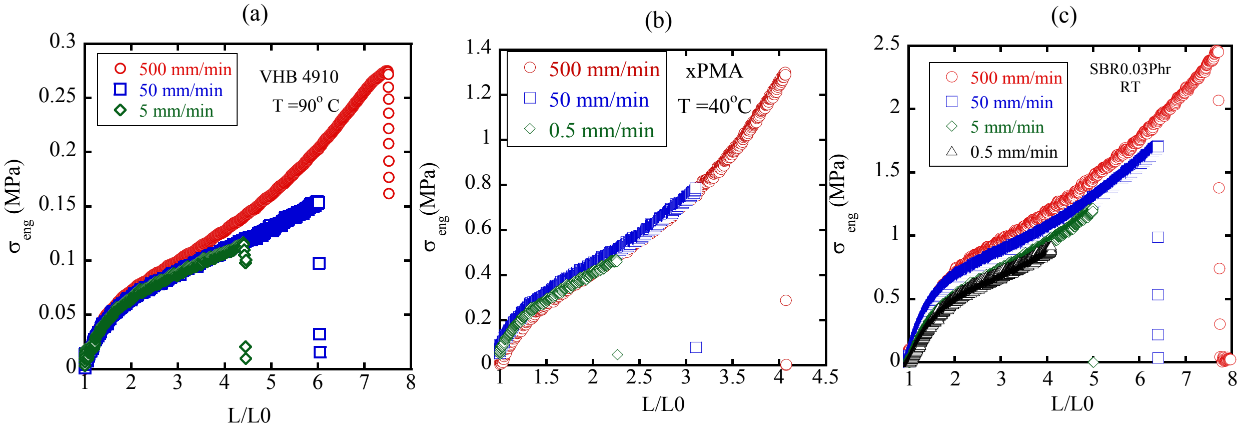

Fig. SI.2 Continuous uniaxial stress strain (SS) curves for three different elastomer samples, demonstrating the effect of crosshead speed on their rupture behavior. The tests were performed on dogbone specimens for: **(a)** VHB at 90 °C; **(b)** xPMA at room temperature; and **(c)** SBR0.03 Phr at room temperature.

Dimensions for the VHB and xPMA dogbone were 63 mm × 3.18 mm × 1 mm with distance between the clamped part of L_0=27 mm, while the custom made SBR specimen measured 27 mm × 1.56 mm × 1.3 mm with distance between the clamp part of L_0=17 mm. For all three materials, the results confirm that an increasing stretch rate leads to a measurable increase in the stretch ratio at break (λ_b).

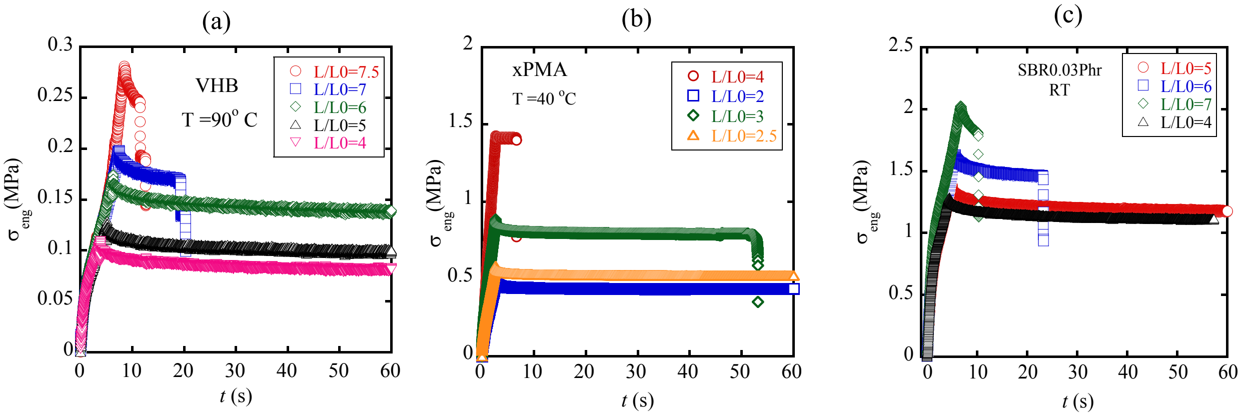

Fig. SI.3 Stress relaxation behavior of three elastomer dogbone samples VHB, xPMA, and SBR0.03 Phr following step strain tests conducted at a constant crosshead speed of 500 mm min^{-1}. The specific conditions for each sample were as follows:**(a)** The VHB sample was tested at 90 °C, with the isometric hold initiated at various draw ratios ranging from 4 to 7.5. **(b)** The xPMA sample was tested at room temperature, with holds initiated at draw ratios between 2 and 4.**(c)** The SBR0.03 Phr sample was also tested at room temperature, with holds initiated at draw ratios from 4 to 7.

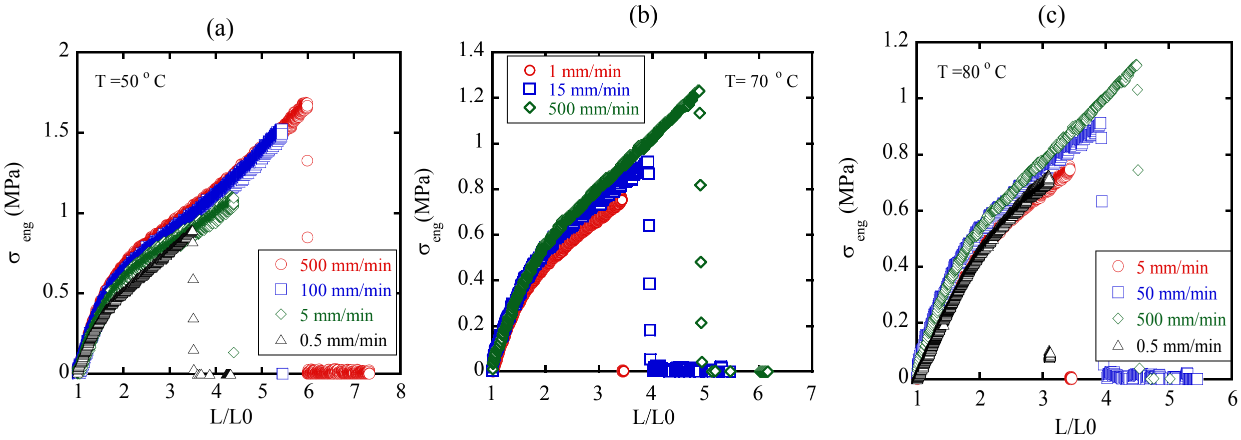

Fig. SI4. Stress strain curves for SBR0.03Phr dogbone samples from continuous stretching tests performed at various crosshead speeds and at elevated temperatures (50, 70, and 80 °C). The experimental data are used to determine the breaking strain and to characterize the rupture time, t_{rupt} as a function of temperature (T) and crosshead speed.
